# Understanding social inclusion: A directed content analysis

**DOI:** 10.1371/journal.pone.0333666

**Published:** 2025-10-03

**Authors:** Yvonne Tan, Imaan Bayoumi, Bruce Knox, Michele Cole, Logan Jackson, Autumn Watson, Colleen M. Davison, Susan A. Bartels, Eva Purkey

**Affiliations:** 1 School of Medicine, Queen’s University, Kingston, Ontario, Canada; 2 Department of Public Health Sciences, Queen’s University, Kingston, Ontario, Canada; 3 Department of Family Medicine, Queen’s University, Kingston, Ontario, Canada; 4 Department of Emergency Medicine, Queen’s University, Kingston, Ontario, Canada; University of Georgia, UNITED STATES OF AMERICA

## Abstract

**Background:**

Social inclusion can be defined as a process that enhances opportunities for social participation, strengthens social bonds, and ensures equitable access to opportunities and decision-making. It emphasizes the interconnectedness of society and the active roles individuals play in upholding shared values and responsibilities. This study aimed to explore factors contributing to social inclusion from the perspective of families in Kingston, Ontario who self-identified as having a history of adversity and as being resilient during the COVID-19 pandemic.

**Methods:**

Participants consisted of a maximum variation sample of families who demonstrated family level resilience during the COVID-19 pandemic. Focus groups and semi-structured in-depth interviews were conducted to allow participants to explore what helped or hindered their family resilience during and beyond the pandemic. Using directed content analysis, line-by-line coding of interview transcripts was conducted to explore the fit between data and an existing social inclusion framework.

**Results:**

The majority of data fit meaningfully into the dimensions established in the social inclusion framework:(1) Quality Education; (2) Innovation & Technology; (3) Governmental Policies & Laws; (4) Transportation & Infrastructure; (5) Employment & Organizations; (6) Poverty & Economy; (7) Medical & Health; and (8) Community & Culture. However, we noted that some dimensions could be both sources of and barriers to social inclusion. Additionally, our study identified specific elements not discussed in the original inclusion framework, including informal education, public gathering spaces, nature, the social dimensions of poverty, and mental health care.

**Conclusions:**

Participants perceived social inclusion as a facilitator of resilience. Interventions targeting the many dimensions of social inclusion must be implemented to drive positive community transformation.

## Introduction

The concept of social inclusion can be defined in many ways. The United Nations Department of Economic and Social Affairs (DESA), defines inclusion as “a multi-dimensional, relational process of increasing opportunities for social participation, enhancing capabilities to fulfill normatively prescribed social roles, broadening social ties of respect and recognition, and at the collective level, enhancing social bonds, cohesion, integration, or solidarity” [1]. Similarly, an inclusive society was defined by the World Summit for Social Development in Copenhagen in 1995 as a society in which “every individual, each with rights and responsibilities, has an active role to play [2].” Other definitions offer a more descriptive understanding of how a society’s parts are interconnected and uphold shared values [3–5]. A commonality in these various definitions is the importance of equitable access to, and participation in, opportunities and decision-making processes.

Some social inclusion frameworks focus on specific populations, such as people with disabilities [6–8], people with dementia [9], and immigrants [10,11]. Others focus on fields such as healthcare [12,13], education [14–16], and technology [17–19]. A framework that captures a more complete picture of social inclusion was developed by Hassan et al. [20] based on a systematic review of the literature on current social inclusion measures. It groups the factors contributing to social inclusion into eight dimensions [20]:

**Table:** 

Dimension	Description
Quality Education	The fair participation of all students without discrimination, allowing for diverse learners to be supported
Innovation & Technology	Equitable access to the internet, social media, online learning resources, and digital data to enhance participation in economic, political, and social lives
Governmental Policies & Laws	The full participation of individuals in political life and the equal distribution of agency, power, and voice.
Transportation & Infrastructure	Transit accessibility, transit policies, and transport amenities, all of which contribute to guaranteeing equal access to goods and services.
Employment & Organizations	The building of positive workplace relationships as well as the ability to have a livelihood.
Poverty & Economy	The ability to reach a minimum standard of living, access financial opportunities and services, and participate in financial systems.
Medical & Health	The opportunity to attain the highest level of individual health and the equitable distribution of medical resources.
Community & Culture	The ability to live in an inclusive community without discriminatory practices.

To further explore the multifaceted nature of social inclusion, this study focused on the lived experiences of individuals and families who have undergone various challenges related to social inclusion. Examining social inclusion through the lens of those who have faced adversity can guide the development of more comprehensive strategies for fostering inclusion, in addition to revealing the strengths and limitations of current frameworks. The following study therefore aimed to explore factors contributing to social inclusion from the perspective of families in Kingston, Ontario who self-identified as having a history of adversity and as being resilient during the COVID-19 pandemic. The framework developed by Hassan et al. was used to guide this analysis.

## Methods

### Design

This analysis is part of a larger study: “Engaging Families to Build Healthy Communities” which was a multiple case study conducted in Kingston, Ontario involving both qualitative and arts-based methods to understand the experiences of families with a history of adversity who self-identified as being resilient during the COVID-19 pandemic. The present study was a secondary analysis of the data to explore the factors contributing specifically to social inclusion for participating families.

“Engaging Families to Build Healthy Communities” was a community based participatory research (CBPR) project guided by critical theory. CBPR serves as a tool for democratic engagement, using co-creation and collaboration in meaningful knowledge production [21]. Community members are involved in each step of the research process, from identifying research questions to data collection, interpretation, and knowledge translation [22]. Critical theory highlights unequal distribution of power and resources as a key factor contributing to existing disparities [23]. Using CBPR guided by critical theory allows for the meaningful prioritization of lived experiences, disrupting traditional research hierarchies to focus on empowering and centering the expertise of participants [21].

Given the qualitative nature of this study, we acknowledge the importance of positionality and reflexivity in shaping the research process. As researchers, we recognize that our perspectives, lived experiences, and institutional affiliations may have influenced data collection, interpretation, and analysis. To manage potential biases, the research team was comprised individuals with varying backgrounds in social sciences, medicine, public health, as well as community researchers (community members from various equity deserving groups with unique lived experiences relevant to the topic of social inclusion). The research team engaged in reflexive practices including reflexive journaling, and well as by collectively reflecting on the data, themes, and results of this study with multiple members considering results to ensure that personal experiences did not unduly bias the results. Additionally, we worked closely with community partners to validate the data, ensuring that knowledge production was collaborative and that participant perspectives remained central to the study.

### Setting and Participants

Recruitment for “Engaging Families to Build Healthy Communities” took place from January 2022 through to March 2023 in the Kingston, Frontenac, Lennox and Addington (KFLA) region. Recruitment was supported by members of the project’s ‘Community Advisory Board’ (CAB), which is made up of representatives from local health and social service organizations and community members with lived experience.

Working with partner organizations on the CAB, participants were invited to participate if they 1) were families, defined as one or more adults acting as primary caregivers for one or more children under the age of 18; 2) self-identified as having experienced adversity, including adverse childhood experiences or adverse community environments; 3) resided in the Kingston, Frontenac, Lennox, & Addington (KFL&A) region; 4) believed their family, or members of their household, would be interested in creating social change for other families like theirs; and 5) could consent for all family members to participate in all components of the study. The use of maximum variation considered factors such as household composition (single parent, two parent household, ages of children), ethnocultural background (newcomer, Indigenous, racialized), and geographic location (urban vs. rural) within KFL&A, among other factors. The goal of these case studies was not to be representative of the local population, but rather to identify rich cases that reflected diverse community experiences and that can inform interventions relevant to other families experiencing adversity. Ten families met inclusion criteria and consented to participate in the study; however, one family withdrew, resulting in a final sample of nine families included in the analysis.

### Data collection

Prior to the study, ethics approval was obtained from the Health Sciences Research Ethics Board (HSREB) at Queen’s University (FMED-6810–21; 6034297). Informed consent was obtained verbally from participants, and voluntary participation and confidentiality were ensured at each stage.

The first step of the primary study involved creating visual timelines and a family focus group discussion. During the visual timeline exercise, families mapped out important life events and experiences that supported or challenged their resilience during the COVID-19 pandemic. The visual timelines were used to facilitate discussions with participating family members where family members were asked about family and community resilience to explore what individual, family, or community factors made it easier or more difficult for them to be resilient. The second step of the study involved individual semi-structured, in-depth interviews with participating family members, including consenting child(ren) 12 years of age or older. Children were interviewed only with the additional consent of their parents and their own assent. These interviews were to allow family members to discuss topics they might not feel comfortable discussing in front of others. The third and final step was a photovoice component. Families were encouraged to take photos of spaces, places, and objects that supported or challenged their resilience. This was followed by semi-structured in-depth interviews using the SHOWeD framework to further explore what helped or hindered their family resilience during and beyond the pandemic [24]. All interviews were audio recorded and transcribed verbatim, and interviews conducted in French were translated into English.

### Data analysis

Directed content analysis was used to explore constructs related to social inclusion. The entire data set from each family was analyzed as one case. In directed content analysis, a framework comprising key themes is identified; these themes are used to establish initial coding categories [25]. Transcripts are then coded according to these categories [25]. Any text that does not fit into this existing coding framework is either assigned a new code or allocated to sub-categories of existing codes [25]. This approach enables the testing and reinforcement of previous theoretical frameworks, in addition to potentially enhancing them [25].

In this study, the framework developed by Hassan et al., described above, was selected as it was felt to provide the most comprehensive understanding of social inclusion across diverse fields. Following framework selection, one researcher (YT) created codes and completed line-by-line coding of the entire dataset using NVivo software. Another researcher (EP) subsequently reviewed the coding process to ensure reliability and consistency.

## Results

Nine families were enrolled in the study. Families ranged from two to five members and included various family compositions, including single parents, reconstituted families, and foster families. Participants included Indigenous, Muslim, newcomer, 2SLGBTQ + , and francophone families. Family members included individuals with a history of substance use, individuals who have experienced homelessness, survivors of sexual violence, individuals living with chronic illnesses, and individuals experiencing mental health issues, amongst others.

### Dimension 1: Quality education

Participants illustrated the importance of quality education in helping enhance their participation in society. Education, and in particular, the formal schooling system, was highlighted as providing students with the opportunity to socialize and feel included. During the pandemic, the lack of in-person schooling was noted to limit children’s ability to interact with others, with one family even noting that the social aspect of getting in trouble in class was missing. Beyond the pandemic, another family shared that their son dropping out of high school limited his inclusion; although he later completed his credits, the greatest difficulty for him was missing milestones such as prom and graduation with his classmates. Such experiences demonstrate that a supportive school environment that allows for students to interact with their peers is a key contributor to social inclusion. For Family 008, a teacher that went above and beyond spending time with struggling students enabled their children to better understand one another. Such quality education breaks down learning barriers and encourages fair participation of all students:

*“I think he had an amazing teacher who kept these kids so engaged. [...] He really brought these 28 students together and every one of them thrived. Every single student he had in his class, every parent said the same thing, they all thrived and they all just came together and they really learnt how to work with each other and respect each other.”* (Family 008)

Education was also highlighted as being a way to learn about inclusion and society at large. For one parent, educational resources at the library – particularly classic North American children’s books such as those by author Dr. Seuss – were seen as a cultural bridge for newcomers like her, helping her and her children more easily integrate into their new environments. Similarly, education about both present-day and historical events was noted by another family as being vital to aligning oneself with the needs of the community; this was highlighted as equipping individuals with the knowledge necessary to engage meaningfully with their communities:

*“I really feel that education is so important. And it does not matter, I don’t mean that you’re graduating high school or you’re graduating elementary school and then going on to further education or becoming a doctor. I mean that you are becoming aware of current events. You’re becoming aware of historical events. You’re becoming in tune with what we need as a community or as a world at large.”* (Family 007)

Furthermore, education equips individuals with access to resources and opportunities, which in turn function to enhance social inclusion. Schools often serve as material support systems for families – the schooling system was highlighted by one family as being crucial to connecting them with a food sharing program. By providing access to resources and networks, schools promote social inclusion by ensuring that families from diverse backgrounds are cared for. Additionally, families highlighted that education provided them with the knowledge and skills to access opportunities such as employment. One participant noted that they wanted to return to school in order to have the opportunity to create a more inclusive community for survivors of sexual violence:

*“[I would like] to be in school and have a good job. [...] I want to work with kids that were like abused when they were little. Like when I was little I was sexually abused. I want to help kids that need to talk about things, you know, get it out there.”* (Family 048)

### Dimension 2: Innovation & technology

Technology was regarded by families as essential to increasing their ability to form connections with others and take part in society. While this was initiated in many cases by the pandemic, it continued as an important tool for inclusion beyond. For example, families noted that technology strengthened existing bonds – many shared that video calls and virtual events allowed them to transcend physical barriers and expand opportunities for meaningful interactions with their families, friends, and local communities:

*“My grandmother tunes into church every weekend and does it all by Zoom call now. So I mean it’s really sort of taught them new skills and allowed them to connect with their community.”* (Family 007)

Several families also expressed that social media and online platforms facilitated new connections and community building among those with shared interests, identities, or experiences. For example, a participant in one family noted that being in a Facebook group for wives that have husbands with cancer enabled her to feel less alone. Another parent created an Instagram account for moms with foster children; the platform was highlighted as a way for her to create a community, participate in discussions, and share information with individuals from around the world:

*“I forged connections with thousands of people all over the world who have gone through similar things. And we’ve been able to be there for each other and that’s been really nice. The cool thing about social media is that you can find people who are experiencing similar things. You’re not limited to your geographical area.”* (Family 009)

Technology was also highlighted as important for increasing the accessibility of resources and opportunities. One parent was able to get their child a comprehensive online assessment for ADHD after being unable to access any in person services in a timely manner. Another parent noted that having an online option for a local library’s community engagement meeting allowed her to participate; she would not have been able to leave her children at home to attend the meeting otherwise. Such access to technology facilitated social participation by enabling her to engage in a community activity that was important to her:

*“And the most important part is that they had an online option for those meetings. So I participated. I wouldn’t have been able to go to the in-person one because it wasn’t at a time that I could attend because [...] it was in the middle of the day or something. But if it’s on Zoom then ok. The kids can be distracted for a bit while I do this. But I can’t leave the house for 4 hours with the kids here.”* (Family 042)

However, it is important to note that technology was not universally experienced as a facilitator of inclusion. Some families expressed a dislike of and lack of privacy during video call therapy, while others experienced anxiety with the use of social media. Several families also noted that a lack of access to a phone and high-speed internet limited inclusion. Additionally, even for those with physical and financial access to technology, a lack of knowledge on how to use different apps and features was a frustration for many, leading to feelings of exclusion. A parent in Family 047 noted that her unfamiliarity with technology led to missed communications with her children’s school and cancelled healthcare appointments:

*“I was feeling like a failure to myself and my kids for not being able to keep up with support via Zoom especially since I had never even sent an email at that point, let alone checked my email daily.”* (Family 047)

### Dimension 3: Governmental policies & laws

Although governmental policies and laws were not a prominent theme contributing to participants’ social inclusion in this study, they were nevertheless discussed by several families. One parent expressed that books teaching her son about activism and democracy were important in helping shape him into a kind and empathetic person, in addition to bringing their community together. Another family highlighted that equitable opportunities to participate in municipal government meetings were important for building strong, inclusive communities where everyone feels valued, supported, and connected:

*“I also learned through that program that there are opportunities for people in poverty to be able to sit on the board in City Council meetings and to impart their knowledge and add to the conversation. And I thought, wow like that’s where we can make some difference. That’s where we can make some impact, some change. [...] The fact that someone like me would even be invited into a conversation was shocking to me.”* (Family 047)

However, negative interactions with governmental policies and laws were noted to limit social inclusion for many. One participant shared that their desire to become a nurse was hindered by their criminal record, while another expressed feeling like an outcast for their hesitancy around vaccines amidst vaccine mandates. More than one family indicated that legal issues and misunderstandings with the Canadian Revenue Agency (the federal agency responsible for administering tax law and policy in Canada) were limiting their participation in society. Several families also noted that the presence of police was either inadequate or actively detrimental to addressing issues of danger and violence in their communities. For Family 043, existing government policies made navigating employment difficult given their status as refugees, which made it challenging to fully participate in and integrate into society:

*“We are neither permanent residents nor temporary residents, which means we are not eligible for many welfare [programs] which are open to Canadians, permanent residents, or temporary residents, like students or workers. [...] I worked part-time, so I was not eligible for Employment Insurance. [...] And I didn’t have a passport until now because the Canadian government detained our passports when we applied for refugee claimant [status]. So it was very hard to explain to employers my circumstances.”* (Family 043)

### Dimension 4: Transportation & Infrastructure

Participants described the importance of accessible transportation in providing access to resources, facilitating social connections, and integrating into their communities. Parents highlighted that public transit – and in particular, a municipal program of free bus passes for youth – was a way for their children to develop autonomy and a sense of self in addition to allowing them to meet up with friends. Conversely, a lack of transportation was noted to limit inclusion – some families noted that unreliable public transit and not having a car left them cut off from community resources, with one family expressing that moving away from a more urban area with frequent buses to Kingston was incredibly isolating. Several families noted that public transit and taxi chits (vouchers) provided by local organizations allowed them to access local community resources and programs while saving time and money; accessible transportation networks enhanced social inclusion for those living in underserved or isolated communities:

*“From where we are right now, it takes us an hour to get from to the [community health centre]. So an hour bus trip down and an hour bus trip back, that’s a huge chunk out of the day. […] To just call a cab and get to where you need to be, quickly and efficiently, and to have that service offered without impacting my budget for groceries – yeah it was a huge life saver.* (Family 047)

Infrastructure and urban planning were also highlighted by many families as being necessary to social inclusion. For example, one family noted that a local community health centre with many resources and programs located in one place provided an effective way of addressing the needs of those who may face barriers to full participation in society, helping them access essential services and opportunities, and fostering a sense of belonging and dignity. Additionally, many participants noted the importance of public gathering spaces. In particular, parks, libraries, community centres, and religious institutions that were geographically accessible were noted to promote social inclusion by bringing people together and providing opportunities for community engagement, learning, and cultural exchange. Such accessible community facilities were especially highlighted as spaces that enabled families to find like-minded individuals with shared interests, values, and aspirations:

*“If I ever go out early, I’m the first to arrive in the park, and people in the neighborhood can see me. It’s visible from the houses and people can join us, children can join us for example. [...] I’d say it’s a meeting place.”* (Family 001)

A key aspect of urban planning that stood out for many families was access to nature. The land played a central role in the cultural and spiritual growth of Family 040; their relationship with the land was noted to be a source of health, knowledge, and spiritual connection. Additionally, access to parks, forests, and other green spaces provided opportunities for those of all ages and backgrounds to engage in outdoor activities. In particular, the importance of ensuring that public infrastructure included access to nature for the entire population was highlighted by many participants. Such access allowed people to connect with others, cultivate mindfulness, and develop coping skills, fostering a sense of belonging in their communities:

*“Making it easy for families and children and everyone to get outside and spend time in nature in a safe way, is so good for mental health. And it’s so accessible to everybody. It’s free. It’s easy to get to.”* (Family 009)

### Dimension 5: Employment & Organizations

Employment and organizations were noted to contribute to social inclusion in a variety of ways. Most evidently, unemployment increased stress and anxiety related to material deprivation in several families, in addition to disruptions in social networks and social support systems. Furthermore, a lack of suitable employment due to factors such as immigration status, pregnancy, lack of access to adequate childcare, or a history of criminal convictions hindered participants’ ability to participate fully in society:

*“I got a full time [job offer] at [thrift store], but they wanted me to push those big cart things while I was 6 months pregnant. So I couldn’t do that. And then [fast food restaurant] wanted me to do overnights.”* (Family 048)

On the other hand, employment was also highlighted as being a source of social inclusion. For many families, access to meaningful work was a source of resilience and fostered a sense of purpose. Employment offered families opportunities for skill development, learning, and personal growth, enabling them to realize their full potential. Moreover, employment was highlighted as empowering families to contribute to their communities, enhancing participants’ sense of belonging:

*“Right now, I am working on [building a homeless shelter]. So I’ve moved from building condos to building projects that help support the community, which has been a great transition.”* (Family 042)

Nevertheless, employment was also a source of stress for many families. For one family, being consumed with long work hours meant that they did not have the time or the capacity to remember to purchase food. For several others, working long hours limited time spent with their children. Additionally, organizations characterized by high workloads, unrealistic expectations, and a lack of support from employers were noted to create stressful work environments, undermining participants’ well-being and leading to feelings of isolation:

*“ My boss called me and told me I was disorganized. She chewed me out on the phone. And I had a panic attack and never went back.”* (Family 007)

### Dimension 6: Poverty & Economy

Poverty was highlighted as limiting social inclusion. Poverty restricts individuals’ access to essential resources such as food and housing, and a lack of access to these resources can hinder individuals’ ability to participate fully in social, economic, and cultural activities. Many families highlighted inadequate living conditions as compromising their health, safety, and dignity, leading to marginalization within society. One family noted that inadequate housing prevented community integration as there was a lack of trust and safety in their neighbourhood:

*“I live in a housing unit. There’s like 50 units in our four corners here and I wouldn’t borrow a cup of milk from 99% of those people because I would never know what I was going to get. So it’s a scary situation. We literally have had a drug bust in a unit that is right here next to us, three times. [...] We live here because we have to, not because we choose to. We just don’t have the income to live in a big fancy house in a fancy neighbourhood.* (Family 008)

Poverty was also noted to widen the gap between socioeconomic groups and erode social cohesion. One family noted that they were treated differently based on the area they lived in; poverty is often accompanied by social stigma, stereotypes, and discrimination. Another family mentioned that, while families with more resources could renovate their basements and their backyards, their children had to use a playground that was duct taped together:

*“I think here in Canada, the people who have more money and more power have access to all the resources. And more towards the bottom would be people who are immigrants or people who maybe don’t have a good income. And they have more disadvantages than people who are at the top.”* (Family 043)

Income was highlighted by many families as being particularly vital to facilitating opportunities for their children. One parent took a second job to pay for her daughter’s ADHD assessment, while another family did not hesitate to spend money on activities for their kids despite worries about the rising cost of living. A third family expressed how being able to participate in extracurriculars facilitated a sense of belonging in their son:

*“My son is 13. He just played soccer for the first time this summer. I’ve never been able to afford it. [...] He was so proud and loved being a part of the team and wore his little jersey to school the next day. He just hasn’t been given the opportunities.”* (Family 047)

### Dimension 7: Medical & Health

Families discussed the impact of health on improving overall quality of life, enabling them to participate fully in individual and community activities. For one participant, being in good physical health allowed them to take on a demanding food delivery job; their health not only enabled them to maintain employment but also provided them with a sense of purpose and contribution to society. Conversely, chronic illnesses, injuries, and disabilities were noted to limit participation in daily tasks, recreational activities, and social interactions, limiting inclusion and well-being:

*“The home-schooling communities that we could be part of are not inclusive enough for us for various reasons. [...] They’re not inclusive for people with disabilities. We finally returned to one of the groups and I can’t be part of it because they go on 10-kilometer hikes where I don’t know what the terrain is and there is no accessibility. I cannot do 10-kilometer hikes without knowing what the terrain is. And I may need help and they have no help available to me. So we had to quit.”* (Family 042)

Mental health was highlighted by many families as contributing to social inclusion. For one participant, therapy to manage their emotions also taught them interpersonal skills, enabling them to navigate challenges, maintain relationships, and foster community. For another participant, having time to recharge on their own allowed them to better contribute to their family and their community. However, poor mental health was noted to be a barrier to inclusion. For several families, issues such as anxiety and substance use disorder led to difficulties interacting with others and social isolation. Additionally, many families had children with mental health issues that limited opportunities for social engagement and participation:

*“Our youngest has sensory processing issues. They likely have FASD and they’re being tested for autism. And it’s hard to go to events or things that are made for “regular families” because they can’t. It doesn’t work for them. It doesn’t work for us. It’s chaotic, it’s stressful, it’s just easier to stay home sometimes.”* (Family 009)

Access to healthcare was described by many families as being essential to their physical and mental health, and thus, their ability to fully participate in society. For one family, an inability to access healthcare in a timely fashion worsened their physical health, leading to worsened chronic pain and increased isolation. For several others, experiences of racism in the healthcare system made them unwilling to return to the hospital and to access Western medicine. Conversely, positive experiences with healthcare resources were noted to be essential to feeling valued and empowered:

*“I’ve dealt with a lot of shame and injustice around being on the methadone program. [...] To be able to have somebody who gets it, who understands addiction specifically and the different complications that can come up with someone who is struggling with addictions and parenting – it was immensely helpful to have that link.”* (Family 047)

### Dimension 8: Community & Culture

Community and culture were noted as being a major source of social inclusion. This theme included family, peers, formal and informal mentorship relationships, community organizations, and civic participation.

Families were a source of both emotional and material support; these forms of support were expressed as promoting social inclusion by providing individuals with validation for their experiences, emotions, and identity in addition to increased economic stability. Participants also consistently acknowledged the importance of caring for their children in helping them to overcome adverse circumstances and stressors, and in turn, fully participate in society. Conversely, a lack of support from family limited inclusion – absent, ill, abusive, or conflictual family members were noted to be barriers to thriving in the community. For one family, a lack of childcare support from their family was a major source of stress:

*“The one thing we struggle with the most is childcare, because we don’t really have a stable environment for them to go to. [...] My mom is in active addiction. And my real dad, he’s around but he has his wife with her kids. [...] Every time we [ask him to watch our kids], he’ll try to avoid it and say - oh, we’re busy this day, and we’re busy today.”* (Family 048)

Peers, including friends, neighbours, and colleagues, were also essential to participants’ experiences of social inclusion. Participants noted that the exchange of assistance, resources, and goods not only helped address immediate needs, but also fostered a sense of interdependence and cultivated a culture of reciprocity in the community. Peers allowed individuals with shared interests, values, and aspirations to foster an environment where they could openly express themselves and feel understood. This in turn allowed for them to feel a sense of belonging, connection, and support:

*“My husband who regularly goes over [to the local Mosque] [...] feels like he belongs, really since, they pray together.”* (Family 001)

Other social networks, including mentors, teachers, and healthcare workers, were also highlighted by participants. One participant’s Master’s program supervisor paid her tuition after she lost her job; this was expressed as being a major contributor to her resilience and ability to continue thriving in the community. Another family had a “street grandmother” who they trusted to take care of their children. Several families also described the importance of teachers who spent extra time with, delivered materials to, and checked in with their children. Relationships with healthcare workers that were longitudinal and non-judgmental were additionally noted to be a source of both tangible support and empowerment, enabling participants to navigate challenges and experience a sense of belonging. For one family, librarians were described as facilitating connections with the rest of the community:

*“One particular librarian moves around between the libraries. And whenever she’s at this one, I’m just so happy because she’s got this gigantic smile. Even with a mask on you can tell. And she’s just so happy to see you all the time. Like no matter what she’s doing, she can look super busy and then she looks up and sees you and just, you know, that huge smile and talks to you and takes the time. And it’s just such a breath of fresh air to have people who are just so connected to whoever they are serving.”* (Family 042)

Families also acknowledged the importance of community services and resources in fostering social inclusion. Indigenous community resources that integrated Indigenous ways of knowing and being were recognized for their importance in contributing to individual, family and community healing, wellbeing, and resilience. Indigenous families expressed a feeling of inclusion in community spaces that reflected Indigenous culture as such spaces created a sense of welcome, safety and understanding. Participants noted that access to Indigenous specific programs and services connected individuals to their culture, in turn contributing to their identity and community belonging. Likewise, services tailored toward women and children provided families with the resources and care needed to navigate their unique challenges. One family noted that the support they received from a centre for mature, female-identifying students proved invaluable in providing herself and her children with practical support, like financial aid and food, along with non-essential resources like holiday gifts. Another notable facility for many families was the library, which served as an accessible resource hub allowing for access to books, games, printers and scanners, and internet. By helping families meet these needs, libraries ensured that families had the foundation for stability, well-being, and social inclusion within their communities. Several families expressed the need for additional community services, including skills training and workshops on topics such as finances and housing, resources for people experiencing homelessness and substance use, and spaces such as community centres, parks, and community kitchens. Additionally, community events and gatherings were desired by many families:

*“More community-based events to let people be familiar with their neighbours [would make me feel safer]. [...] Not city based events like big events where you don’t know each other but you go there. But like if the city or whatever organizations can develop more community-based events and let people in the community get to know each other better and provide a platform for us to get to know each other.”* (Family 043)

Civic participation, or reciprocal participation in their own community, was another source of social inclusion. By engaging in activities such as community service, advocacy, and volunteerism, participants developed a sense of commitment to the needs of the community; this promoted social inclusion by empowering individuals to contribute to positive social change and address shared challenges:

*“Through giving back and volunteering, I would say that also just makes me feel like I’m contributing to the community as a whole and not just taking. Like we have taken so much it feels like from all of the different counselling that we have received, that it feels nice just to reinvest in the community.”* (Family 007)

Nevertheless, components of community also served as a barrier to social inclusion for some families. Public displays of substance use were expressed by several participants as making them feel unsafe, limiting their freedom of movement and hindering community engagement. Additionally, intimate partner violence caused some participants to experience stigma, shame, and self-blame, leading to social isolation. Discrimination was noted by participants as leading to social fragmentation, leading to a lack of a sense of belonging in the community and reinforcing divisions and inequalities within communities:

*“I was struggling with my mental health and so I was looking for religious support. So I was looking for that spiritual connection. But I guess in 7 years I forgot what churches are like. And so we walked out in the middle of a very transphobic sermon and will not go back. That was very traumatic and I shouldn’t say disappointing because I should have known. But uh it was disappointing because like that is a great way to build community.”* (Family 009)

## Discussion

Among families who self-identified as having a history of adversity but who were also identified as resilient, social inclusion was consistently discussed and factors contributing to social inclusion echoed existing inclusion frameworks. An absence of social inclusion due to barriers or absence of resources (e.g., technology, finances, etc.) was felt to present a challenge to families’ ability to be resilient, whereas when social inclusion was felt, families identified that this improved their resilience. While all families in our study perceived themselves to be resilient, not all families attained a high degree of social inclusion. Nevertheless, all identified that they believed that greater social inclusion either did, or would have had it been present, enhance their resilience. Mapping these factors to such a framework provides an important lens through which the complex construct of social inclusion can be understood; as such, this analysis can support policymakers at municipal, provincial and federal levels, community organizations, and service providers in determining how social inclusion can be addressed.

Our study also identified specific elements not discussed in the original inclusion framework. These included informal education, public gathering spaces, nature, the social dimensions of poverty, and mental health care.

Similar to our findings, previous studies have highlighted the ways in which different constructs contribute to social inclusion. Quality education has been noted to help connect diverse students and facilitates inclusion [16,26,27]. Likewise, technology has been highlighted as an important means of enhancing community participation for various groups, including those with disabilities, those living in rural areas, and older adults [28–31]. Governmental policies and laws that enable all citizens to access essential services have been found to enhance social inclusion [32,33], while well-functioning transportation systems and adequate infrastructure have been shown to increase daily activity participation and social cohesion [34–36]. Additionally, several studies have noted that unemployment and labour market insecurities are important barriers to inclusion [37–40], and that increasing poverty and income inequality have exacerbated the marginalization of vulnerable groups [41–43]. The availability of affordable healthcare within responsive health systems has been found to directly impact social inclusion [44,45], while living in a community free from discrimination has been noted to increase social participation and inclusion [46,47].

The alignment of our study’s findings with existing literature underscores the critical need to address the various dimensions contributing to social inclusion. We propose the following recommendations:

1. Universal access to digital services and training:

Participants identified how technology could be a barrier or a facilitator to inclusion. Our study is not the first to recommend universal access to the internet (including hardware and software) as well as universal training in digital literacy as an increasingly important human right linked to an individual’s right to freedom of expression, association, and assembly. Additionally, intergenerational initiatives to enhance digital inclusion can help make technology more accessible, furthering its ability to act as a facilitator of social inclusion [30,48]. We recommend that this access be facilitated as a coordinated effort between different levels of government.

1. Improved access, coordination, and delivery of culturally safe healthcare:

We recommend that at a time when provincial governments across Canada are struggling with ever increasing health care crises, increasing government investment in strengthening the availability, coordination and communication between health delivery systems, including primary care and mental healthcare in community as well as in hospital can enhance social inclusion through healthcare. Additionally, intentionally ensuring training of health care workers in anti-discrimination and cultural competencies, and facilitating effective dialogue between community and health care providers are recognized as potential solutions to improving access and social inclusion through health systems [45].

1. Enhanced, community informed municipal infrastructure:

Our study echoed other findings which indicate benefits related to social inclusion of improved accessibility of public transit and infrastructure, including to green spaces and nature. Governmental policies and laws promoting opportunities to participate in decision making [49], as well as investments in transit and social infrastructure [34,50,51] may alleviate existing inequalities and reduce the risks of social exclusion. In order to strengthen social support networks, opportunities for physical, social and creative community recreation (including parks, green spaces, and community centres among others) should be developed, designed and implemented in coordination with community members, and located in communities where families live [52].

Additional recommendations from our study include literacy programs for vulnerable adults, transition programs for newcomers, and access to volunteer and job resources for older adults which have been found to improve both employment and social inclusion [40,53,54]. Likewise, in an era of increasing food insecurity, difficulties accessing appropriate housing and employment, financial inclusion and well-constructed social policies have been found to contribute to more equitable economic growth, reduce poverty, and promote income equality by providing access to formal financial services and reducing feelings of stigma and shame [43,55]. In implementing such interventions, it will be important to take a participatory approach that allows families facing adversity to develop and build solutions that help them thrive. Centering these voices will enable policymakers to better understand the unique needs and potential barriers faced by these communities in local contexts.

A strength of the study was the use of community based participatory research, allowing for collaboration, reflection, and mutual learning between stakeholders. Spending time and building trust with each family allowed the study team to elicit information that would not otherwise have been available from families experiencing adversity. A potential limitation of the study was the relatively small sample of families, including an absence of representation from rural families. It is possible that factors such as transportation would have been more prominent for families living in rural areas who are dependent on private transportation. Additionally, it is important to note that the values and ideas of social inclusion discussed in this study are largely derived from a Eurowestern colonial framework, which do not fully encompass the diverse cultural realities of Indigenous community members [56].

## Conclusion

Fostering social inclusion is essential for building equitable and resilient communities, particularly for those affected by individual and structural adversity. Although our overarching study was not primarily focused on the construct of inclusion, when asked what helped or hindered family resilience during and beyond the pandemic, participants consistently highlighted that the presence or absence of factors contributing to social inclusion were significant facilitators or barriers to their ability to be resilient, and these factors mirrored existing inclusion frameworks. This reaffirms the importance of inclusion as a determinant of wellbeing, in addition to elucidating the complex dimensions contributing to the concept of social inclusion.

Future studies conducted across different cities and in families facing different social contexts, as well as studies on the effectiveness of specific interventions to enhance families’ overall sense of inclusion are warranted. Ultimately, the journey towards a more inclusive society is ongoing, but concerted efforts and collaboration at the individual, community, and policy levels can drive positive transformation within communities.
